# Investigating the Effect of Processing and Material Parameters of Alginate Dialdehyde-Gelatin (ADA-GEL)-Based Hydrogels on Stiffness by XGB Machine Learning Model

**DOI:** 10.3390/bioengineering11050415

**Published:** 2024-04-24

**Authors:** Duygu Ege, Aldo R. Boccaccini

**Affiliations:** 1 Institute of Biomaterials, Department of Materials Science and Engineering, University of Erlangen-Nuremberg, 91058 Erlangen, Germany; aldo.boccaccini@fau.de; 2 Institute of Biomedical Engineering, Bogazici University, Rasathane St., Kandilli, 34684 İstanbul, Turkey

**Keywords:** machine learning, ADA-GEL, osteochondral regeneration, XGBoost algorithm

## Abstract

To address the limitations of alginate and gelatin as separate hydrogels, partially oxidized alginate, alginate dialdehyde (ADA), is usually combined with gelatin to prepare ADA-GEL hydrogels. These hydrogels offer tunable properties, controllable degradation, and suitable stiffness for 3D bioprinting and tissue engineering applications. Several processing variables affect the final properties of the hydrogel, including degree of oxidation, gelatin content and type of crosslinking agent. In addition, in 3D-printed structures, pore size and the possible addition of a filler to make a hydrogel composite also affect the final physical and biological properties. This study utilized datasets from 13 research papers, encompassing 33 unique combinations of ADA concentration, gelatin concentration, CaCl_2_ and microbial transglutaminase (mTG) concentrations (as crosslinkers), pore size, bioactive glass (BG) filler content, and one identified target property of the hydrogels, stiffness, utilizing the Extreme Boost (XGB) machine learning algorithm to create a predictive model for understanding the combined influence of these parameters on hydrogel stiffness. The stiffness of ADA-GEL hydrogels is notably affected by the ADA to GEL ratio, and higher gelatin content for different ADA gel concentrations weakens the scaffold, likely due to the presence of unbound gelatin. Pore size and the inclusion of a BG particulate filler also have a significant impact on stiffness; smaller pore sizes and higher BG content lead to increased stiffness. The optimization of ADA-GEL composition and the inclusion of BG fillers are key determinants to tailor the stiffness of these 3D printed hydrogels, as found by the analysis of the available data.

## 1. Introduction

Natural polymeric hydrogels such as gelatin and alginate are commonly studied for tissue engineering applications and are being increasingly considered in 3D bioprinting approaches [1,2,3,4,5]. Alginate is an FDA-approved biopolymer exhibiting biocompatibility, hydrophilicity and high water absorption capacity [1,6]. Due to its remarkable properties, alginate (sodium salt of alginic acid) is a widely studied component of bioinks [7,8]. However, alginate-based hydrogels have some disadvantages for 3D bioprinting [9,10,11]. For example, alginate has relatively low viscosity which leads to a low printing accuracy [12,13]. Additionally, alginate hydrogels have uncontrolled degradation kinetics [7,8]. Moreover, as alginate does not contain any adhesive molecular ligand to enable cell attachment, other biopolymers, ideally protein-based, can be combined with it to enable a more effective cell–material interaction [2,7,14]. An alginate derivative produced by controlled chemical oxidation of sodium alginate from brown algae is oxidized alginate (alginate dialdehyde, ADA) [15,16,17]. Dialdehyde groups form in the alginate backbone by the oxidative transformation of the hydroxyl groups at positions C-2 and C-3 [18,19]. ADA-based hydrogels have been shown to be attractive matrices for vascular, bone, cartilage, and wound healing applications [20,21,22,23,24]. 

Gelatin is produced by breaking the triple helix of collagen fibrils by basic or acidic hydrolysis [7,25,26]. Gelatin incorporates cell adhesion peptides through the RGD (Arg-Gly-Asp) collagen sequence, which improves cell–material interactions and enhances cell adhesion and proliferation [2,8]. However, gelatin has poor mechanical properties [1,2,27]. To address the drawbacks of alginate and gelatin taken separately, the combination of partially oxidized alginate and gelatin (ADA-GEL) hydrogels is being increasingly investigated for 3D bioprinting [15,28,29]. Gelatin contains ε-amino groups of lysine and hydroxylysine, which are covalently bonded to the generated aldehyde groups of ADA through Schiff’s base formation [7,8,30]. The ADA-GEL system offers a wide range of tunable properties such as controlled degradation and hydrogel stiffness [31,32]. Moreover, ADA-GEL hydrogels have been shown to support cellular (e.g., osteoblasts and fibroblast cells) activities [1,33].

Transglutaminases are a class of enzymes, present both inside the human body and externally, responsible for catalyzing the formation of ε(γ-glutamyl) lysine isopeptide bonds among protein side chains [34,35,36]. Microbial transglutaminase (mTG) is a specific type of transglutaminase produced by bacteria, notably *Streptoverticillium mobaraense* and *Streptoverticillium ladakanum* [37,38,39]. Unlike some other transglutaminases, mTG functions independently of calcium and has been utilized in various applications, including the fabrication of gelatin scaffolds [40,41]. One of the remarkable characteristics of mTG is its ability to crosslink ADA-GEL hydrogels [15]. This crosslinking process allows for the fine-tuning of hydrogel properties, such as degradation rate, stiffness, and promotion of cell attachment. Notably, this method has proven effective for improving the stability and mechanical properties of gelatin-based materials, addressing the issue of rapid degradation often associated with a high gelatin content [7,15]. Importantly, the crosslinking approach facilitates a precise control over hydrogel stiffness within a broad range, from less than 5 kPa to as high as 120 kPa [15]. To enhance the mechanical properties and biological activity of hydrogels, often rigid inorganic particles, such as calcium phosphate and bioactive glass (BG) particles, are added to the hydrogel forming composites. Such composites can be also applied in 3D (bio)printing [42,43,44].

Since the application of ADA-GEL hydrogels in 3D bioprinting involves numerous variables, it is challenging to fully understand how each parameter taken independently impacts the resulting properties of the printed structure. The Extreme Gradient Boosting (XGB) machine learning technique is an easy-to-use technique with fast performance and high accuracy. Generally, it prevents overfitting and works well with small datasets [45,46]. However, it is prone to overfitting if not well tuned, and hypertuning may be time consuming [45,47]. This study aims to enhance the prediction of mechanical properties, in particular the stiffness of ADA-GEL 3D printed constructs reducing the need for extensive trial and error when preparing such ADA-GEL hydrogel structures. The research examined the importance of BG filler content, ADA to GEL concentration ratio, mTG and CaCl2 concentration (as cross-linkers), pore size, and the established correlation of such parameter through a heatmap to assess their relationship with stiffness. Then, in detail, the effect of ADA concentration, CaCl_2_ concentration, pore size, and filler (BG) content on stiffness was examined. 

## 2. Methodology

### 2.1. Data Collection

A search from 2000 to 2024 was performed with the search engines Web of Science, Scopus, and Google Scholar. The search terms were as follows: ADA-GEL, oxidized alginate, gelatin, mTG, CaCl_2_, stiffness, modulus, and mechanical properties. ADA, gelatin, CaCl_2_, mTG, scaffold pore size, BG filler content, and whether biomaterials were printed or not were used as independent variables while the measured stiffness was used as the dependent variable from 13 research papers, which were finally used by the XGB algorithm [1,2,3,4,5,6,7,8,9,10,11,12,13]. Overall, this dataset covered 33 possible combinations of the independent variables in set ranges of ADA concentration (2.5–7.5 *w*/*v*), gelatin concentration (2.5–7.5 *w*/*v*), CaCl_2_ concentration (0.1–0.6 *w*/*v*), (mTG) concentration (0–10 *w*/*v*), pore size (0–4000 µm), filler content (0–5 wt%), and stiffness (0–417 kPa). Table 1 shows the printing parameters and Table S1 (presented in the supplementary file) shows all processing conditions and resultant stiffness values.

In other studies, ADA-GEL scaffolds were not printed but their stiffness was evaluated [31,43,51].

### 2.2. Computational Modeling

Python programming language is utilized for data analysis and machine learning [52]. Phyton has Pandas, Numpy, and Scipy libraries have tools for data manipulation, numerical computing, and scientific computing, respectively. Additionally, Matplotlib and Seaborn libraries were used for data visualization. Finally, Scikit-learn was used for utilizing XGB algorithm for data analysis [53,54]. The Python codes which are utilized in the study are given at https://github.com/duyguege/machine-learning.git (accessed on 30 November 2023).

#### 2.2.1. XGB Regressor

XGB regressor is an ensemble gradient boosting algorithm which was developed by Guestrin and Chen in 2016 [55,56]. In this model, after evaluation of previous trees, sequential trees are added [57]. This way, a strong learner is developed by training weak learners [45]. The predictions are made by adding up the score of each leaf node [58]. XGB is a scalable tree boosting system with great efficiency and prediction accuracy, that is often employed in the field of regression. To avoid over-fitting to outliers, XGB applies second-order Taylor expansion to the loss function, and normalisation to the objective function [45,55,56,59,60,61,62].

#### 2.2.2. Training, Hyper Tuning and Validation Processes

The dataset was divided into training (80%) and test (20%) divisions for this investigation. The training dataset was used to develop the model, and the test dataset was used to evaluate it. The Python Scikit-learn library was used to apply the models. After determining the optimal parameters for the XGB model, hyperparameter tuning was performed for the subsample ratio of columns, number of estimators, maximum depth, and learning rate (shrinkage factor), as shown in Table 2. To avoid underfitting and overfitting, the optimum parameter for both training and test sets was chosen [60]. For performance evaluation, 10-fold cross-validation was utilized, which randomly divides the dataset into ten equal folds [63]. The model was then used again in the final stage to get the expected stiffness values [64].

#### 2.2.3. Correlation Heatmap

A correlation heatmap visually represents the correlations between multiple variables in a dataset. This graphical tool demonstrates patterns and relationships within complex datasets. It uses colors to indicate the strength and direction of these correlations, with warmer colors (orange) indicating stronger positive correlations and cooler colors (purple) indicating weaker or negative correlations [65]. The Seaborn module of Phyton generated a correlation heatmap to identify the strength of the association between the seven independent factors (ADA and GEL concentration, CaCl_2_ and mTG concentrations, pore size, whether the biomaterial is printed, filler (BG) content), and the dependent variable (stiffness). Additionally, a greater correlation coefficient (r) for different variables suggests that the independent variables are multicollinear [66].

#### 2.2.4. Feature Importance

Feature importance analysis ranks the influential factors within a dataset for gaining insights into the key variables [67]. The significance of the features was calculated using an integrated function in the Scikit-learn implementation of the XGB model. The features were then ranked in order of importance [68,69].

#### 2.2.5. Determination of the Model Performance

Higher R^2^ values with lower mean absolute error (MAE) values show that the models are more likely to succeed [70,71]. To assess model performance, the coefficient of determination (R^2^), MAE calculation, and RMSE were utilised [72].

#### 2.2.6. Shapley Additive Explanation

In 2017, Lundberg and Lee [73] introduced the Shapley additive explanation (SHAP) technique, which allows for the study of complex relationships in machine learning models. SHAP, a model interpretation tool in Phyton, is used in this work to further understand the marginal link between predicted stiffness value and each feature. SHAP value is calculated as the average forecast produced with each feature value’s contribution minus the prediction made without a feature value. A negative or positive SHAP value implies that the feature value has a negative or positive contribution to the prediction of stiffness, respectively. The SHAP summary plot is used to show the impact of each parameter on stiffness. The primary y-axis displays the SHAP value, while the secondary y-axis shows a color bar displaying the high feature values.

## 3. Results and Discussion

**Figure 1:**
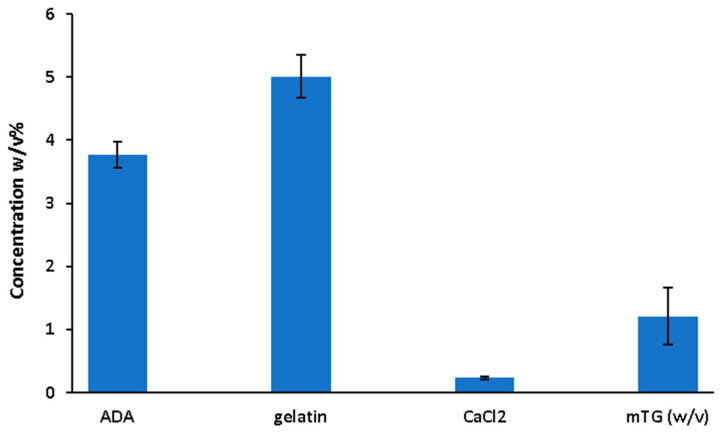
Average of used parameters used in the model.

In this study, factors affecting the stiffness of ADA-GEL composites are evaluated and then predictions are made by using XGB modeling. This approach is useful to evaluate the complex relationships between processing parameters and stiffness in a snapshot. Therefore, the study may support the design of future experimental investigations using ADA-GEL-based hydrogels. Figure 1 shows the average concentration of each independent variable used in the reviewed papers. For the independent variables, constraints would occur due to different factors, such as high viscosity or fluidity of the hydrogels, which influence the 3D printing process. In the study, the parameters used were constrained within the range found in the literature.

**Figure 2:**
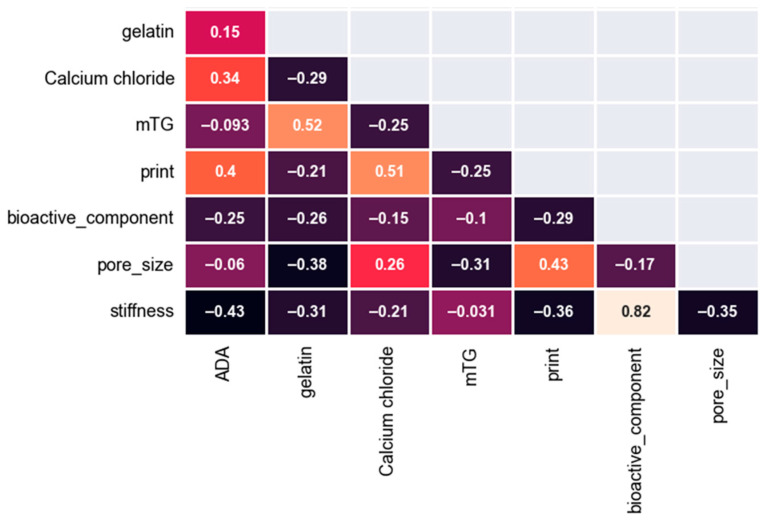
Correlation heatmap for the independent and dependent variables.

Figure 2 shows the correlation heatmap for all of the considered variables.

A correlation coefficient exceeding 0.8 signifies a very strong correlation, while a coefficient between 0.6 and 0.8 suggests a good correlation. Within the range of 0.4 to 0.6, a moderate correlation is observed, whereas a coefficient between 0.2 and 0.4 demonstrates a comparatively weak correlation. Values below 0.2 indicate an extremely weak correlation [66]. Additionally, negative coefficients indicate an inverse correlation, with the same interpretation applying to the strength of these correlations [74]. As shown in the correlation heatmap, there is a negative but weak correlation between printing and stiffness. This indicates that printing leads to reduced stiffness compared to hydrogel scaffolds which are produced by casting. Firstly, layer-by-layer deposition is prone to weakened interlayer adhesion [75]. The data available thus indicate that 3D printing conditions should be improved to produce scaffolds with enhanced mechanical performance. Pore size also has a negative correlation with mechanical stiffness; when pore size increases, the mechanical properties, in this case stiffness, deteriorate. BG fillers have a high and positive correlation of (0.82), which shows a positive effect of the presence of BG filler on stiffness. The relative effect of gelatin, ADA and CaCl_2_ concentrations, which show relatively weak correlation with stiffness, will be analyzed in detail in this paper. On the other hand, mTG has a very small correlation coefficient of −0.031 and this could be indicated as an extremely weak correlation with stiffness.

**Figure 3:**
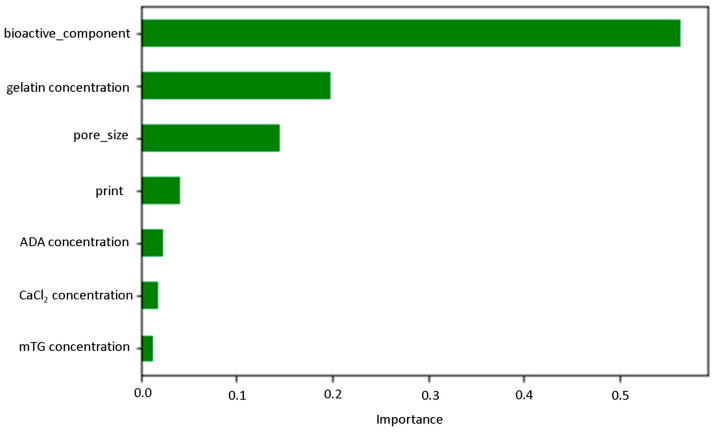
Feature importance score of the studied parameters.

Another important aspect to consider is multicollinearity between different variables. Lack of identification of multicollinearity may lead to misinterpretation of data [76,77]. If there is a high correlation coefficient for different independent variables, this may indicate multicollinearity [76]. In this study, independent variables have low correlation coefficients between themselves. The highest correlation coefficients were between mTG and gelatin concentration (0.52) and CaCl_2_ concentration and printing (0.51), respectively, however, it is still relatively a low value and these parameters can be considered relatively independent [76]. Therefore, it can be stated that multicollinearity was not found in the dataset considered for this study. Figure 3 shows the feature importance of the independent variables.

**Figure 4:**
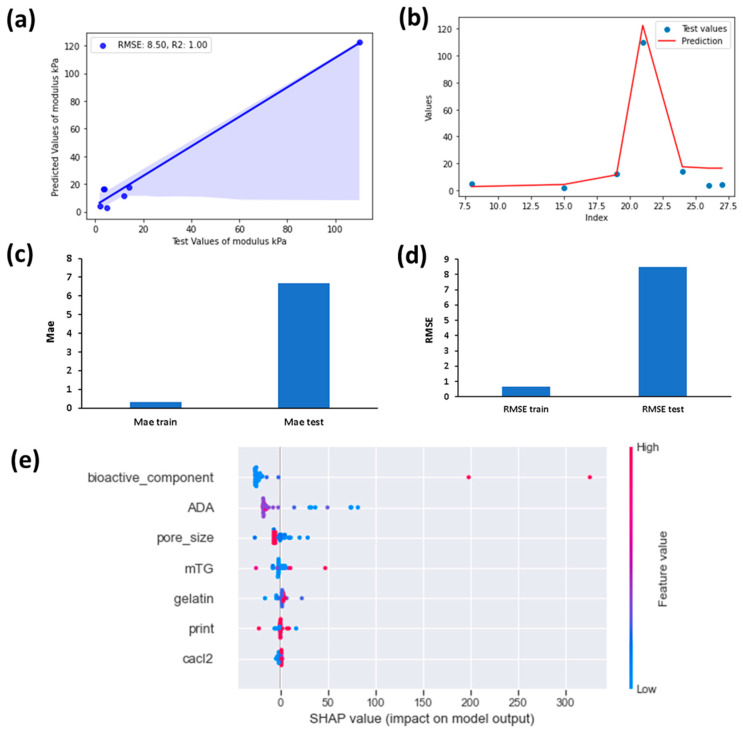
Determination of the success of the model. (**a**) Predicted and observed stiffness values for XGB, (**b**) distribution of experimental (test) and predicted values with XGB, (**c**) MAE train and test values, (**d**) RMSE train and test values, (**e**) SHAP summary plot based on XGB model for feature values.

Feature importance analysis illustrates the most influential factors in predictive modeling, providing valuable insights into key variables impacting model outcomes. It is known that understanding these influential parameters enhances model interpretability and guides decision-making processes [68,69]. According to Figure 3, the addition of BG filler has a very high impact on the stiffness of ADA-GEL composites, following with the concentration of gelatin and the pore size. The printing, ADA content, CaCl_2_ and mTG concentrations have a much weaker effect on stiffness. In Figure 4, the success rate of the XGB model for predicting stiffness from the provided data is shown.

The R^2^ value is an important indicator of the success of the prediction [70,71]. A value of 0.6 or below indicates an unsatisfactory prediction. A value between 0.6 and 0.75 proves a satisfactory prediction. A value between 0.75 and 0.95 shows a good prediction and a value above 0.95 means an excellent prediction [78]. According to Figure 4, XGB can predict the test values well, which is confirmed by R^2^ value of 1, which indicates an excellent prediction. However, according to Figure 4b,c, there is overfitting of the data for test values. In Figure 4b, the fitting of the predicted values to the test values is illustrated for different test values, which is indicated by their index values from the dataset. According to the figure, the fitting demonstrated a superior fitting for some of the test values, while others (index between 25 and 27) showed inferior fitting. Figure 4c,d supports this observation from Figure 4b. This is apparent from the low train values for both RMSE and MAE, however, much higher RMSE and MAE values were found for the test data. This probably occurs due to the relatively small sample size. The discrepancy between the R^2^ value and RMSE/MAE may also arise if the range of observed values in the dataset is limited. In such cases, the model may appear to fit the data perfectly, but the absolute errors between the predicted and observed values are still relatively high, leading to a high RMSE/MAE [79]. This problem could be solved if a larger sample size could be obtained.

**Figure 5:**
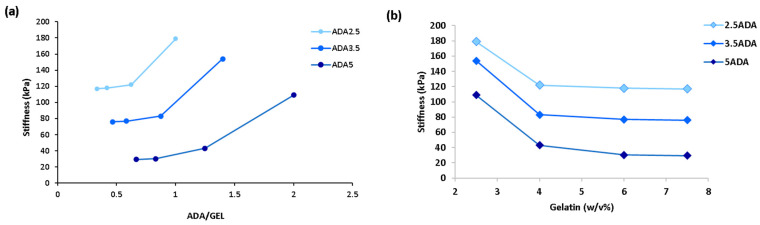
Predicted stiffness of ADA-GEL hydrogels for different compositions: (**a**) predicted stiffness vs. ADA/GEL content, (**b**) predicted stiffness vs. gelatin concentration for various ADA concentrations (CaCl_2_ = 0.1 *w*/*v*, mTG = 1 *w*/*v*%, filler content = 1 wt% and pore size = 200 µm).

A SHAP summary plot illustrates the contribution of each feature to the model’s output [80]. According to Figure 4e, it is clearly observed that lower concentrations of BG filler component reduced stiffness values and higher BG content improved the stiffness. According to Figure 4e, high SHAP values lead to higher predictions of SHAP value, whereas low SHAP values reduce the predicted value. Blue colour is used for small values and red colour is used for high values of the variables. According to Figure 4e), low concentrations of ADA supported the model to make high predictions of stiffness. On the other hand, usually high ADA values lead to negative SHAP values which indicate that it had a negative impact on stiffness. Low pore size values also led to higher predicted values as expected, and higher values led to decrease of stiffness. Due to reduced resistance to deformation, as pore size decreases, fewer voids form and less variability occurs in the microstructure. This leads to less scattering of the material properties and higher mechanical properties can be achieved [81,82,83]. Lower BG content led to lower stiffness predictions and higher BG content led to higher stiffness predictions. For mTG, CaCl_2_ and gelatin content, no such distinct relationship could be observed from the SHAP values, as high values and low values of the dataset are distributed more randomly. After analyzing the data with SHAP summary, predictions were made for different variables to analyze their influence on stiffness. In Figure 5, the predictions made are shown for different concentrations of ADA and gelatin.

**Figure 6:**
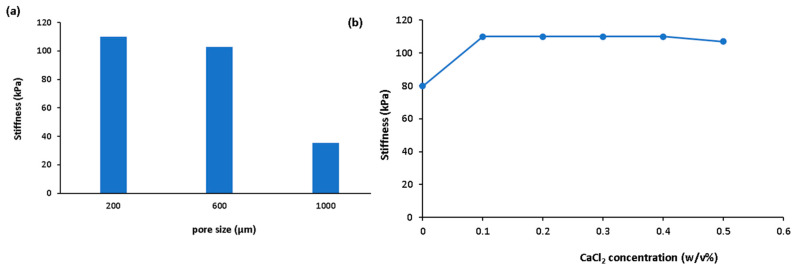
Change of predicted stiffness with (**a**) pore size (ADA %*w*/*v* is kept as 3.75, gelatin = 2.5, CaCl_2_ = 0.1 *w*/*v*, mTG = 1 *w*/*v*%, filler content = 1 wt%) and (**b**) CaCl_2_ concentration (ADA %*w*/*v* is kept as 3.75, gelatin = 2.5, mTG = 1 *w*/*v*%, filler content = 1 wt%, pore size = 400 µm).

As can be seen from Figure 5a, as the ADA/GEL content increases, the stiffness increases. When the concentration of ADA is higher than that of gelatin, usually a higher stiffness is achieved. Figure 5b shows that when the *w*/*v*% of gelatin increases, the stiffness decreases, which is likely because some of the present gelatin cannot covalently crosslink with aldehyde groups of ADA. Unbound gelatin in the hydrogel leads to a weaker scaffold. A similar explanation has been previously made in the studies of Distler et al. [15] and Sarker et al. [43] for the effect of ADA/GEL content ratios on stiffness. For instance, when gelatin content increases from 2.6 to 7.5% *w*/*v*% for 5 *w*/*v*% ADA, the stiffness reduces from 60 kPa to 20 kPa. This reduction is quite abrupt, and this result proves clearly that high gelatin content reduces the mechanical performance. ADA content also significantly affects stiffness. Lower ADA content systematically led to higher stiffness values in the predictions in Figure 5. This aligns well with the SHAP summary plot for ADA dataset which indicated that lower ADA values led to higher stiffness (higher SHAP values). According to Figure 5, 2.5 *w*/*v*% ADA with 2.5 *w*/*v*% GEL leads to the highest stiffness of 200 kPa among study groups. However, some of these ADA/GEL concentration ratios are not experimentally practiced and therefore require experimentation to determine their applicability. Figure 6 shows the effect of pore size and CaCl_2_ concentration on stiffness.

Pore diameters of 3D scaffolds ranging from 20 to 500 µm are critical in tissue engineering because they allow for cell ingrowth, bone regeneration, and vascularization [48]. The optimal pore size for collagen fibre production was reported to be in the nanometric range (100 nm), whereas the pore size required for cell seeding, migration, and distribution is in the range of 100 µm to mm depending on the cell type. A size of 100 µm is appropriate for chondrocytes [7]. The literature shows that addition of gelatin to ADA can reduce the pore size and the addition of BG can also further reduce pore size [43]. A balance of pore size and mechanical properties is required to achieve tissue regeneration. It has been reported that an ideal pore size of 300 µm may enable both suitable mechanical performance and tissue regeneration [43]. In principle, according to Figure 6, a pore size in the range 200–400 µm appears appropriate to maintain suitable mechanical properties. The elastic modulus of mammalian chondral tissue (matrix surrounding chondrocytes) (25 ± 5 kPa) is much lower than that of bone (cortical bone 15 ± 5 GPa). Brain tissue has a stiffness of approximately ~1 kPa. For example, for cartilage regeneration, 200 µm pore size would enable suitable mechanical performance [31,48]. It is possible to modulate the mechanical performance of the ADA-GEL hydrogels by varying the relative ADA and gelatin concentrations. Crosslinker concentration is also important for obtaining desired mechanical properties. For alginate, CaCl_2_ is the most commonly used crosslinking agent [84]. In Figure 6b, CaCl_2_ concentration was found to lead to an optimum stiffness with a concentration of 0.1 *w*/*v*%. With further increase of CaCl_2_, no apparent change of stiffness was obtained. 

**Figure 7:**
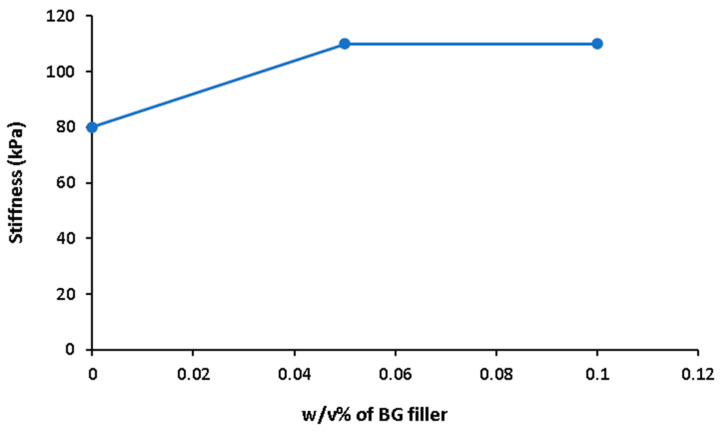
Effect of *w*/*v*% of BG filler on predicted stiffness of ADA-GEL composite hydrogels (ADA = 3.75 wt/v%, gelatin = 2.5 wt/v%, CaCl_2_ = 0.1 *w*/*v*%, mTG = 1 *w*/*v*%, pore size = 400 µm).

Figure 7 shows the effect of BG filler on stiffness. In some studies, BG fillers are blended in ADA-GEL not only to enhance hydrogel bioactivity, but also to increase the mechanical properties and the stability of the hydrogel in physiological conditions [43]. BG fillers support gelation and increase the crosslinking degree of ADA-GEL [42]. In a previous study, Sarker et al. [43] showed that the gelation time of ADA-GEL decreased in the presence of BG. According to XGB model, there is an increase of stiffness with the increase of *w*/*v*% of BG in the hydrogels. However, higher *w*/*v*% of BG leads to hydrogels that cannot be printed due to the increase of viscosity, which limits the *w*/*v*% of BG to 0.1. Moreover, in this study, mTG concentration effects were not considered. mTG was usually used as 1 *w*/*v*% in the literature and high concentrations of mTG may be toxic, therefore in the predictions mTG was kept as 1 *w*/*w*% [85]. 

A limitation of this study arises from the diverse range of techniques employed to assess the stiffness of the hydrogels. The methods of measurement include nanoindentation, compression test, and dynamic mechanical analysis [20]. This may lead to differences of measured values of stiffness due to wide variation of conditions used in the different tests. Additionally, in some studies, gelatin has been heat-treated previously to its use in making the ADA-GEL, however, this could not be taken in consideration as it could drastically reduce the dataset’s sample size [48]. Moreover, 3D printed scaffolds had varying numbers of layers, which may also affect the measured stiffness as well as different printing speed and printing pressure applied for printing the scaffolds. Another important parameter determining the stiffness of ADA-GEL is the degree of oxidation of alginate, which may ultimately affect the mechanical properties. However, as can be seen from Table 1, this parameter is not provided in some of the research papers, therefore we could not incorporate it into the model. Finally, in some studies, 3D printed scaffolds were cell laden while in others, they were not. It is reported in the literature that cell seeding density significantly impacts the mechanical properties of alginate hydrogels [86]. As can be seen from Table S1, in the study by Kara et al. [40], 3D printed MC3T3-E1 cell laden ADA-GEL (3.75–7.5%) scaffolds were produced and the stiffness values were found to be relatively lower (10 kPa) than the 3D printed ADA-GEL scaffolds without cells. Furthermore, Schwarz et al. [7] 3D printed chondrocyte laden ADA-GEL (3.75–3.75%) scaffolds and a stiffness value of 27 kPa was achieved, which was also found to be relatively low. In the the future, it would be beneficial to concentrate on these parameters in order to more precisely control the mechanical properties of printed scaffolds. Furthermore, changes of molecular weight among different studies would also lead to inaccurate predictions and need to be taken into consideration, however, this information is not provided in all the papers [47]. 

In the studies, the used bioactive glass also showed variation. It was not possible to find enough datasets for the same type of bioactive glass to be incorporated to the algorithm. However, in all studies, bioactive glasses were observed to positively impact stiffness. Therefore, this parameter was incorporated to the study. In the future, it would be beneficial to study the effect of only a single type of bioactive glass on the mechanical properties of ADA-GEL, to have a more precise outcome. If these limitations could be addressed, and a larger sample size could be obtained, the model might not exhibit overfitting which was illustrated in Figure 4b,c. 

The data analysis for stiffness of ADA-GEL is not sufficient to fully comprehend the hydrogel mechanical performance. Unfortunately, in the literature there is not sufficient data on compressive strength, % strain at break, and toughness of these hydrogels to build models for these features. These are also important parameters to be considered while evaluating the appropriateness of these hydrogel-based scaffolds for tissue engineering applications. Nevertheless, this preliminary study can guide the identification of additional study groups. This could facilitate the inclusion of a larger sample size in future machine learning models to re-analyze ADA-GEL hydrogels for tissue engineering applications. Finally, another important and heavily investigated class of hydrogels for biofabrication and tissue engineering is gelatin methacrylate (GelMA) [87,88,89]. We anticipate that a much larger dataset exists for GelMA-based hydrogels than for ADA/GEL. In the future, a similar model can be also applied to GelMA (or other heavily exploited biomaterials) yo analyze their mechanical properties as a result of combination of determined processing variables.

## 4. Conclusions

In this study, the XGB model was used to study the effects of various preparation parameters, including BG filler content, ADA/GEL concentration ratio, pore size, mTG and CaCl_2_ concentration (as cross-linkers) on the stiffness of ADA/GEL hydrogel composites. This research emphasized the impact of the ADA/GEL concentration ratio, indicating the critical role of the ADA component in modulating hydrogel stiffness. Additionally, the gelatin component showed an inverse correlation with stiffness as higher gelatin concentrations led consistently to decreased stiffness. SHAP analysis further indicated that lower ADA concentrations increased the predicted value of stiffness. Additionally, it was identified that the pore size of the printed scaffolds (with 200–400 µm pore diameters) led to an ideal balance for mechanical performance and suitable pore size for applications in tissue engineering. The incorporation of BG fillers demonstrated a significant increase in ADA/GEL composite stiffness, providing a potential option for enhancing hydrogel stability and mechanical properties. In conclusion, this study provides insight into the effects of key processing and material parameters on ADA-GEL hydrogel composite stiffness in a snapshot. This knowledge is useful for researchers to fine-tune these parameters for specific tissue engineering applications. 

## Figures and Tables

**Table 1:** Printing parameters used in the relevant papers [1,2,7,8,40,42,48,49,50].

Printing Temperature (°C)	Printing Speed (mm/s)	Pressure (kPa)	Ref.
30	N/A	N/A	[42]
30	2	160	[48]
30	5	165	[2]
30	N/A	8	[1]
30	10	250	[49]
30	N/A	N/A	[50]
30	14	35	[8]
30	10	100	[40]

**Table 2:** Hyperparameters and their best parameters for tuning by XGB model.

Hyperparameters	Subsample Ratio of Columns	Number of Estimators	Maximum Depth	Learning Rate
Parameters	0.5, 0.6, 0.8, 0.9	1000, 2000	4, 6, 10	0.01, 0.05, 0.1, 0.3
Best parameters	0.8	1000	4	0.3

## Data Availability

The original contributions presented in the study are included in the article/supplementary material, further inquiries can be directed to the corresponding author.

## Supplementary Materials

The following supporting information can be downloaded at: https://www.mdpi.com/article/10.3390/bioengineering11050415/s1, Table S1: Composition, crosslinker concentrations and physical properties of the prepared hydrogels [1,2,7,8,15,31,40,42,43,48,49,50,51].
