# Supplementary material for: Investigating the Effect of Processing and Material Parameters of Alginate Dialdehyde-Gelatin (ADA-GEL)-Based Hydrogels on Stiffness by XGB Machine Learning Model
Source: Bioengineering (Basel). 2024 Apr 24;11(5):415. doi: 10.3390/bioengineering11050415 (PMC11117982; doi:10.3390/bioengineering11050415)
Supplement: Supplementary file 1 [file bioengineering-11-00415-s001.zip › bioengineering-2905837-supplementary.pdf]

bioengineering

MDPI

Supplementary

Table S1. Composition, crosslinker concentrations and physical properties of the prepared hydrogels [1,2,7,8,15,31,40,42,43,48–51].

Indices	ADA (w/v)	Gelatin (w/v)	with/without cells (printed/not printed)	$CaCl_2$ (M)	mTG (w/v)	Pore size (µm)	Bioactive component	Degree of oxidation	Stiffness (kPa)	Ref.
1	2.5	2.5	Without cells (not printed)	0.1	0	450	0	35	135	[43]
2	2.5	2.5	Without cells (not printed)	0.1	0	450	1	60	304	[43]
3	2.5	2.5	Without cells (not printed)	0.1	0	450	5	75	417	[43]
4	3.35	6.65	Without cells (Not printed)	0.1	5	500	0	N/A	13	[51]
5	2.5	3.75	Without cells (printed)	0.2	0	500	0	N/A	10	[42]
6	2.5	3.75	Without cells (printed)	0.2	0	500	0.1	N/A	50	[42]
7	3.75	3.75	Without cells (printed)	0.1	0	4000	0	N/A	4.2	[48]
8	3.75	3.75	Without cells (printed)	0.1	0	4000	0	N/A	3.9	[48]
9	3.75	3.75	Without cells (printed)	0.1	0	4000	0	N/A	5	[48]
10	3.75	3.75	Without cells (printed)	0.1	0	4000	0	N/A	2	[48]
11	3.75	3.75	Without cells (printed)	0.1	0	4000	0	N/A	1	[48]
12	3.75	3.75	Without cells (printed)	0.5	0	4000	0	N/A	5	[48]
13	3.75	3.75	Without cells (printed)	0.5	0	4000	0	N/A	4.5	[48]
14	3.75	3.75	Without cells (printed)	0.5	0	4000	0	N/A	5.4	[48]
15	3.75	3.75	Without cells (printed)	0.5	0	4000	0	N/A	2.5	[48]
16	3.75	3.75	Without cells (printed)	0.5	0	4000	0	N/A	1.9	[48]
17	3.75	3.75	Chondrocyte laden (printed)	0.4	2.5	217	0	N/A	24	[7]
18	3.75	7.5	Chondrocyte laden (printed)	0.4	2.5	217	0	N/A	35	[7]
19	7.5	3.75	Without cells (printed)	0.5	0	1000	0	N/A	11.5	[1]
20	7.5	3.75	Without cells (printed)	0.5	0	1000	0	N/A	12	[1]
21	3.575	7.5	Without cells (printed)	0.1	2.5	1000	0	N/A	100	[49]

22	2.5	3.75	Without cells (printed)	0.6	0	1000	0	13	110	[50]
23	2.5	3.75	Without cells (printed)	0.1	0	1000	0	N/A	135	[8]
24	3.75	7.5	MC3T3-E1 cell laden (printed)	0.1	10	370	0	N/A	10	[40]
25	3.75	7.5	Without cells (printed)	0.1	0	1000	0	N/A	4	[15]
26	3.75	7.5	Without cells (printed)	0.1	0.1	1000	0	N/A	3.5	[15]
27	3.75	7.5	Without cells (printed)	0.1	1	1000	0	N/A	4	[15]
28	3.75	7.5	Without cells (printed)	0.1	2.5	1000	0	N/A	10	[15]
29	3.75	7.5	Without cells (printed)	0.1	5	1000	0	N/A	25	[15]
30	3.75	7.5	Without cells (printed)	0.1	10	1000	0	N/A	100	[15]
31	2.5	2.5	hiPSC (not printed)	0.1	0	1000	0	19	17	[31]
32	5	7.5	Without cells (not printed)	0.2	0	1000	0	N/A	14	[2]
33	5	7.5	Without cells (not printed)	0.2	0	1000	0.1	N/A	22.5	[2]
